# Micropattern Silk Fibroin Film Facilitates Tendon Repair *In Vivo* and Promotes Tenogenic Differentiation of Tendon Stem/Progenitor Cells through the *α*2*β*1/FAK/PI3K/AKT Signaling Pathway *In Vitro*

**DOI:** 10.1155/2023/2915826

**Published:** 2023-01-13

**Authors:** Kang Lu, Hong Tang, Yang Wang, Liyuan Wang, Mei Zhou, Gang He, Hao Lu, Chuyue Tang, Wan Chen, Xiaoqing Ma, Kanglai Tang, Zhongliang Deng

**Affiliations:** ^1^Department of Orthopedics-Spine Surgery Center, the Second Affiliated Hospital, Chongqing Medical University, Chongqing, China; ^2^Department of Orthopedics/Sports Medicine Center, State Key Laboratory of Trauma, Burn, and Combined Injury, Southwest Hospital, Army Medical University (Third Military Medical University), Chongqing, China; ^3^State Key Laboratory of New Materials Composite Technology, Wuhan University of Technology, Wuhan, Hubei, China; ^4^School of Chemistry and Chemical Engineering, Yangtze Normal University, Chongqing, China

## Abstract

**Background:**

Tendon injuries are common clinical disorders. Due to the limited regeneration ability of tendons, tissue engineering technology is often used as an adjuvant treatment. This study explored the molecular pathways underlying micropattern SF film-regulated TSPC propensity and their repairing effects to highlight the application value of micropattern SF films.

**Methods:**

First, we characterized the physical properties of the micropattern SF films and explored their repairing effects on the injured tendons *in vivo*. Then, we seeded TSPCs on SF films *in vitro* and determined the micropattern SF film-induced gene expression and activation of signaling pathways in TSPCs through high-throughput RNA sequencing and proteomics assays.

**Results:**

The results of *in vivo* studies suggested that micropattern SF films can promote remodeling of the injured tendon. In addition, immunohistochemistry (IHC) results showed that tendon marker genes were significantly increased in the micropattern SF film repair group. Transcriptomic and proteomic analyses demonstrated that micropattern SF film-induced genes and proteins in TSPCs were mainly enriched in the focal adhesion kinase (FAK)/actin and phosphoinositide 3-kinase (PI3K)/AKT pathways. Western blot analysis showed that the expression of integrins *α*2*β*1, tenascin-C (TNC), and tenomodulin (TNMD) and the phosphorylation of AKT were significantly increased in the micropattern SF film group, which could be abrogated by applying PI3K/AKT inhibitors.

**Conclusion:**

Micropattern SF films modified by water annealing can promote remodeling of the injured tendon *in vivo* and regulate the tendon differentiation of TSPCs through the *α*2*β*1/FAK/PI3K/AKT signaling pathway *in vitro*. Therefore, they have great medical value in tendon repair.

## 1. Introduction

Improper exercise, accidents, and aging have increased the incidences of tendon injuries [1–3]. Putting aside unreported cases, more than 30 million tendon injuries occur worldwide every year, which causes inconvenient living conditions and financial pressure to patients [4–6]. Tendon injuries are typically treated with physical therapy or surgery (e.g., allogeneic tendon transplantation). However, scar healing as well as low mechanical strength and tissue adhesion remain common clinical pitfalls resulting from the existing treatments [6–8]. Therefore, new tendon repair techniques with better curative outcomes are needed.

The application of silk fibroin- (SF-) based biomaterials has been widely used for the repair of bone, cartilage, tendon, and other tissues due to their remarkable advantages, including high strength, elasticity, excellent biocompatibility, low toxicity, and easy processing [9–11]. In addition, SF can be produced in the forms of membranes, fibers, particles, and scaffolds to meet there pair demands of different tissues [12–14]. Enhanced cell propensities and biological behaviors have been shown when cells are grown on a material with a similar physical surface micropattern as their tissue of origin [15–17]. Thus, bionics has emerged as a new direction in the design and preparation of biomaterials for tissue repair [14, 18–20]. In our previous studies, we demonstrated that SF films mimicking the physical surface micropattern of fiber morphology of native tendon collagen can promote the tenogenic differentiation of tendon stem/progenitor cells (TSPCs) [21, 22]. However, the repairing effect of micropattern SF films in tendon injury and their effect on regulating the intrinsic TSPC signaling pathways remain unclear.

*In vivo* studies are essential for assessing the repairing effects of biomaterials [23–25]. Tendon is a type of dense connective tissue, and evaluation of the reparative effect of the injured tendon includes both noninvasive and invasive methods, such as magnetic resonance imaging (MRI) and histological examination [26, 27]. High-throughput omics technologies such as RNA sequencing and proteomics are potent methods for characterizing altered cellular signaling pathways [28–30].

In this study, we evaluated the repairing effect of the micropattern SF films in the rat Achilles tendon injury model and explored the molecular mechanisms underlying the micropattern SF-mediated tendon repair though bioinformatics analysis. Our *in vivo* tendon repair experiment showed that transplantation of TSPCs cultured in micropattern SF films significantly promotes the reconstruction of collagen fibers and thus recovery of the injured tendons. Mechanistically, the micropattern SF film activates focal adhesion kinase (FAK) and phosphoinositide 3-kinase (PI3K)/AKT signaling pathways and induces the expression of tendon repair marker genes scleraxis (SCX), tenascin-C (TNC), tenomodulin (TNMD), and collagen type 1 alpha 1 (COLIA1) in TSPCs. The results in this study highlight the potential therapeutic application of micropattern SF films in tendon repair.

## 2. Materials and Methods

### 2.1. Preparation and Characterization of SF Films

SF films were prepared as previously described [21, 22]. Briefly, the SF solution was dried to form smooth or micropattern films on polydimethylsiloxane (PDMS) plate, and their stability was improved by water-annealing treatment. Morphological structure of the surface of the SF films was observed by scanning electron microscopy (SEM) and atomic force microscopy (AFM). The SF film samples were further sputter coated with gold for 60 s and observed by SEM (Phenom ProX, Phenom, Netherlands) at 15 kV. The SF samples, before and after modification, were cut into small flakes and observed under AFM (MFP-3D Infinity; Asylum Research, Buckinghamshire, UK). The protein secondary structures in SF films of different groups were analyzed by the Fourier-transform infrared spectroscopy (FTIR; Nicolet Nexus670; Thermo Fisher Scientific, Rockford, IL, USA).

### 2.2. The Rat Achilles Tendon Defect Repair Model

All procedures and *in vivo* experiments were in accordance with the standard guide approved by the ethics committee of Army Medical University (Chongqing, China) and performed according to the guidelines of the National Institutes of Health for the Care and Use of Laboratory Animals. Fifty male Sprague-Dawley's rats (180–200 g, from Southwest Hospital Animal Center (Chongqing, China) were used in this study and randomly assigned to the following five groups: N, control group; D, defect group; R, injury replacement group; S, smooth SF film group; G, micropattern SF film group.

**Figure 1:**
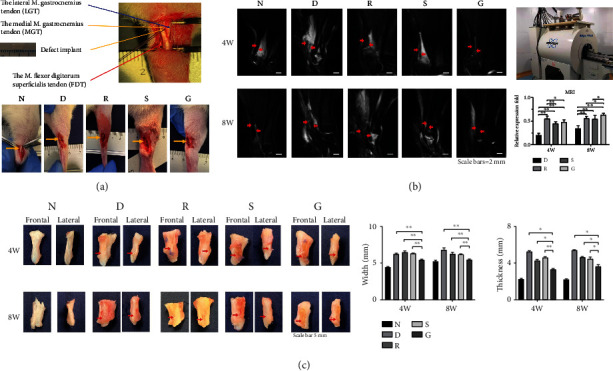
The Achilles tendon defect model and macroscopic repair assessment. (a) Schematic diagram of the anatomy of the rat Achilles tendon and comparison of postoperative surgical areas in different repair groups. Group N: normal tendon (control group); Group D: defect suture group; Group R: allogeneic replacement repair group; Group S: smooth SF film repair group; Group G: micropattern SF film repair group. (b) MRI comparison of tendon repair after 4 and 8 weeks. The red arrow indicates the repaired area, and the repair signal intensity in group G was significantly higher than that in other groups at 8 weeks. (c) Comparison of the width and thickness of the Achilles tendon repair samples. The width and thickness of Group G were significantly lower than those of the other repair groups.

SF films were prepared according to a method described in our previous study [21], and then the SF films were cocultured with TSPCs for 2 weeks before implantation. The rats were anesthetized by intraperitoneal injection of pentobarbital, and the amount of anesthetic was based on rat weight. As shown in Figure 1(a), the medial gastrocnemius tendon of the right hind limb was selected as the surgical site. The right hind limb was shaved and disinfected with povidone iodine, and a 20 mm longitudinal incision was made in the skin to expose the Achilles tendon. The surgical methods of each group were as follows: Group N, sutured layer by layer after exposing the Achilles tendon; Group D, a 3 mm full-size defect of medial gastrocnemius tendon was created; Group R, after preparing the defect like Group D, the tendon removed from Group D was implanted into the defect site, and then a nylon 4-0 suture (FS249-10 pcs, Beyotime, Shanghai, China) was used to fix both ends; Group S, after preparing the defect like Group D, the smooth SF film-TPSC construct was transplanted to the defect site; Group G, after preparing the defect like Group D, the micropattern SF film-TPSC construct was transplanted to the defect site. The skin incisions were closed using nylon 3-0 sutures (Jinhuan, China). The animals were given free access to food and water. Relevant tests were performed in the 4^th^ and 8^th^ week after surgery.

### 2.3. MRI

All MRI scans of the rat Achilles tendons were obtained with the BioSpec 70/20USR MRI system (Bruker, Ettlingen, Germany) postoperatively, at 4 and 8 weeks for five rats from each group. The sequence performed using the surface coil was sagittal T2-weighted imaging. ImageJ software was used to compare the repair signal (low-intensity signal) of the tendon area to semiquantitatively evaluate the repair effect in the different groups.

### 2.4. Histomorphometry

Tissue specimens (*n* = 3 per group) collected at the indicated time points were fixed in 10% formaldehyde at room temperature for 24 h and dehydrated over an alcohol gradient before embedding in paraffin. Prepare longitudinal sections (5 *μ*m thick) and stain them with hematoxylin and eosin (H&E) according to the manufacturer's protocol. The modified histological scoring system (Table S1) from a previous study was used to assess the quality of regenerated tissue [28]. The photomicrographs of all parts were captured in the suture area. Three randomly selected fields of view of each slice were examined under an optical microscope (Olympus, Tokyo, Japan).

### 2.5. IHC

A series of 3 *μ*m-thick sections (*n* = 3 per group) were utilized for IHC. The expression of tendon repair markers in slice samples was observed with an inverted fluorescence microscope (IX71; Olympus). The following primary antibodies were used: anti-TNC (1: 100, bs-1039R), anti-COLIA1 (1: 200, bs-10423R), anti-SCXA antibody (1: 100, bs-12364R), and anti-TNMD antibody (1: 100, bs-7525R); all primary antibodies were obtained from Bioss (Beijing, China). The horseradish peroxidase-conjugated goat anti-rabbit antibody was used as secondary antibody (1: 200, GB23303; Servicebio, Wuhan, China). The images of were analyzed by ImageJ software.

### 2.6. Cell Culture and Grouping

The cell culturing was prepared as follows: TSPCs in Group N were cultured in 6-well plates, and TSPCs in Group G were cultured on SF films with micropatterns. The preparation process and related coculture details have been reported in our previous studies [21, 22]. In brief, we poured the SF solution on a customized PDMS substrate and dried it to form a micropatterned SF film. After water annealing and sterilization, TSPCs were planted on the surface. In Dulbecco's modified Eagle's medium (DMEM; Gibco, Carlsbad, CA, USA), the cells were cultured in DMEM supplemented with 10% fetal bovine serum, penicillin (100 U/mL)/streptomycin (100 mg/mL), and L-glutamine (2 mmol/L) (all were obtained from Invitrogen, Carlsbad, CA, USA). The culture environment was 37°C with 5% CO_2_, and the medium was changed every 3 days.

### 2.7. Bioinformatics Analysis

Bioinformatics analysis in this study included transcriptomics and proteomics analyses, which were completed by Gene Biology (Shanghai, China). In brief, the samples were prepared as follows. After 14 days of culture, the TSPCs in Groups N and G were trypsinized, followed by washing with phosphate-buffered saline (PBS). Total RNAs were extracted with TRIzol Reagent (Takara Bio, Shiga, Japan), and the samples were stored at -80°C before RNA sequencing. Genes with an adjusted *P* < 0.05 found by DESeq2 were considered differentially expressed. Gene Ontology (GO) enrichment analysis of differentially expressed genes (DEGs) was implemented by the cluster Profiler R package, in which gene length bias was corrected. GO terms with corrected *P* < 0.05 were considered significantly enriched by DEGs. We used the cluster Profiler R package to test the statistical enrichment of DEGs in Kyoto Encyclopedia of Genes and Genomes (KEGG) pathways.

For proteomics, TSPCs in Groups N and G were washed three times with PBS and scraped off with cell scraper. Then, the samples were suspended in PBS and transferred to a centrifuge tube. The supernatant was removed after centrifuging at 1000 rpm/min for 5 min, and the remaining TSPC samples were quickly frozen in liquid nitrogen and stored at -80°C before proteomics analysis. The spectrometry data were analyzed using the Max Quant software version 1.6.14.0. The cutoff of global FDR for peptide and protein identification was set to 0.01. Protein abundance was calculated on the basis of the normalized spectral protein intensity. Proteins with fold change > 2 or <0.5 and *P* < 0.05 (Student's *t*-test) were differentially expressed proteins (DEPs). The Fisher exact test was used to enrich GO terms by comparing the number of DEPs and total proteins correlated to GO terms. KEGG pathway analysis was performed using the KEGG database. Fisher's exact test was used to identify the significantly enriched pathways by comparing the number of DEPs and total proteins correlated to pathways.

### 2.8. Western Blot Analysis

After 7 and 14 days of culture, the TSPCs in Groups N and G with (N+ and G+) or without (N- and G-) phosphoinositide 3-kinase (PI3K)/AKT pathway inhibitor (LY 294002; Bioss) were lysed in lysis buffer containing a mixture of proteinase inhibitors (Thermo Fisher Scientific). Equal amounts of protein samples (30 *μ*g/lane) were resolved by sodium dodecyl sulfate-polyacrylamide gel electrophoresis and electrotransferred to polyvinylidene difluoride membranes. Then, the membranes were incubated overnight at 4°C with the following primary antibodies: anti-TNC (1: 1000, ab108930; Abcam, Cambridge, UK), anti-TNMD (1: 1000, ab203676; Abcam), anti-AKT (1: 1000, 9272S; Cell Signaling Technology (CST), Danvers, MA, USA), anti-phospho-AKT (pAKT473, 1: 1000, 9271S; CST), anti-phospho-AKT (Thr308) (pAKT308, 1: 1000, 9275S; CST), anti-PIK3CA (PI3K, 1: 1000, bs-2067R; Bioss), anti-phospho-PI3KCA (p-PI3K, 1 : 1000, bs-5570R; Bioss), anti-integrin *α*2 (1 : 1000, bs-52613R; Bioss), anti-integrin *β*1 (1 : 1000, bs-0486R; Bioss), and anti-GAPDH (1 : 1000, ab8245, Abcam). GAPDH was used as a loading control. Then, the membranes were incubated with secondary antibody (10285-1-AP, 1 : 2000; Proteintech, Wuhan, China) for 2 h at room temperature. The protein bands were visualized by enhanced chemiluminescence (GE Healthcare, Wuxi, China), and the results were analyzed with ImageJ software.

### 2.9. Immunofluorescence

After 7 days of culture, the TSPCs in the different groups were fixed in 4% paraformaldehyde and washed three times with PBS. The fixed cells were incubated with primary antibody overnight at 4°C, followed by incubation with secondary antibody for 90 min at room temperature. Nuclei were stained with DAPI for 10 min. The following primary and secondary antibodies were used: anti-integrin *α*2 (1 : 1000, bs-52613R; Bioss), anti-integrin *β*1 (1 : 1000, bs-0486R; Bioss), and Cy3-conjugated Affinipure Goat Anti-Rabbit IgG (1 : 100, SA00009-2; Proteintech, Wuhan, China). Samples were observed with a laser scanning confocal microscope (LSM880; Carl Zeiss, Jena, Germany). The mean fluorescence intensity was analyzed by ZEN software (blue edition 2.3; Carl Zeiss). Three different images for each group were used for statistical analyses.

### 2.10. Statistical Analyses

Statistical analyses were performed using SPSS 26.0. The results are presented as the mean ± standard deviation. Data were analyzed using analysis of variance and independent samples *t*-test. *P* < 0.05 was considered statistically significant.

## 3. Results

### 3.1. Characterization of the SF Films

**Figure 2:**
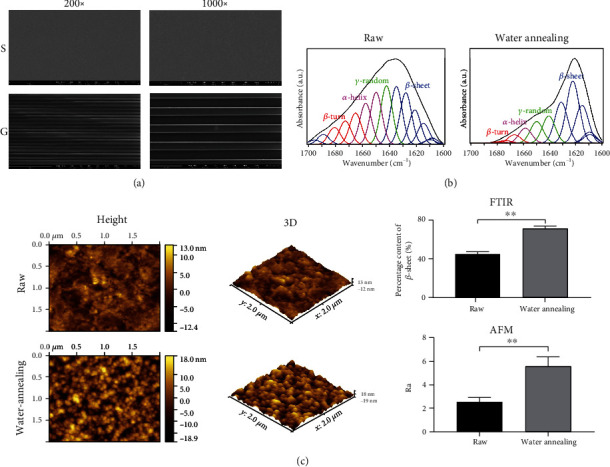
SEM, FTIR, and AFM characterization of SF films. (a) SEM. Group S: SF films without micropatterns; Group G: SF film with micropatterns. The surface of SF film material was clear and stable. (b) FTIR. The *β*-sheet content in micropattern SF film after water annealing was significantly increased. (c) AFM. The nanoscale roughness on the surface of the micropattern SF film after water annealing treatment was significantly increased.

As shown by SEM, the surface morphology of the SF films was clear and stable, making them suitable for cell and animal experiments (Figure 2(a)). The FTIR results displayed a *β*-sheet secondary structure, which represented the stability of the SF films; it was significantly increased after water annealing (Figure 2(b)). In addition, the results of AFM suggested that the surface roughness of micropattern SF films was significantly increased after water-annealing modified (Figure 2(c)).

### 3.2. MRI

We assessed the extent of tendon repair at the injury site by MRI. As shown in Figure 1(b), the existing and nascent tendon tissues exhibited a black hypointense signal while the inflamed areas displayed a white hyperintense signal in the MRI scans. Quantification of the intensity of the black signal indicated that the recovery degree of the R group was significantly higher than that of the other groups in the early stage of repair (4 weeks), whereas the recovery extent of the G group was highest in the later stage of repair (8 weeks).

### 3.3. Macroscopic Morphology

As shown in Figure 1(c), the tendons in Group D exhibited apparent inflammatory hyperplasia with the repaired tendon tissues being significantly wider and thicker than that in other groups. Instead, the nascent tendons in Group G were of a smaller width and thickness than those in Groups R and S. The inflammatory hyperplasia of callus in Group G was less than that in other groups.

### 3.4. H&E Staining and Histological Scoring

**Figure 3:**
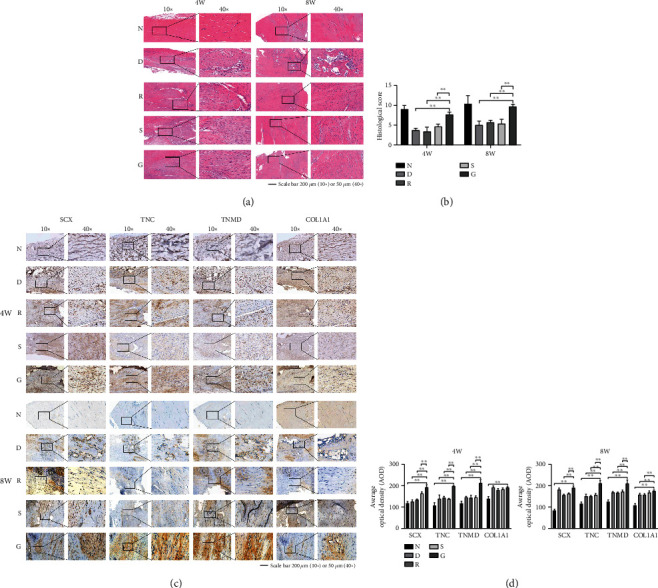
Comparison of histological evaluation of different repair groups. (a) H&E staining. (b) Histological score. The repair score of Group G was higher than that in the other repair groups. (c) Results of IHC. (d) Statistics of IHC results. The tenogenic markers in Group G were significantly higher than those in the other repair groups.

Next, we assessed the extracellular matrix (ECM) remodeling effect in different groups at 4- and 8-week post injury by histology. As shown in Figure 3(a), the tendons in Group D were dominated by scar tissue, with disordered collagen fibers and a large number of infiltrated inflammatory cells. In Group R, despite the formation of nascent tendon tissue, the fiber structure was not continuous with the original fibers at the injury site. A small amount of continuous fibers was found in the regenerated tendons in Group S, whereas the micropattern SF films significantly promoted the formation of collagen fibers at 8 weeks in Group G. A quantified assessment according to the histological scoring table (Table S1) showed that repaired tendons in Group G had the highest score, indicating the best condition of reconstruction of collagen fibers at the injury site (Figure 3(b)).

### 3.5. IHC

As shown in Figures 3(c) and 3(d), IHC showed that the expression of tendon repair markers SCX, TNC, TNMD, and COLIA1 was low in Group N. There was no significant difference in the expression of these marker genes among Groups D, R, and S. The expression levels of all tendon markers were highest in Group G.

### 3.6. Analyses of Differential mRNA and Protein Expression

**Figure 4:**
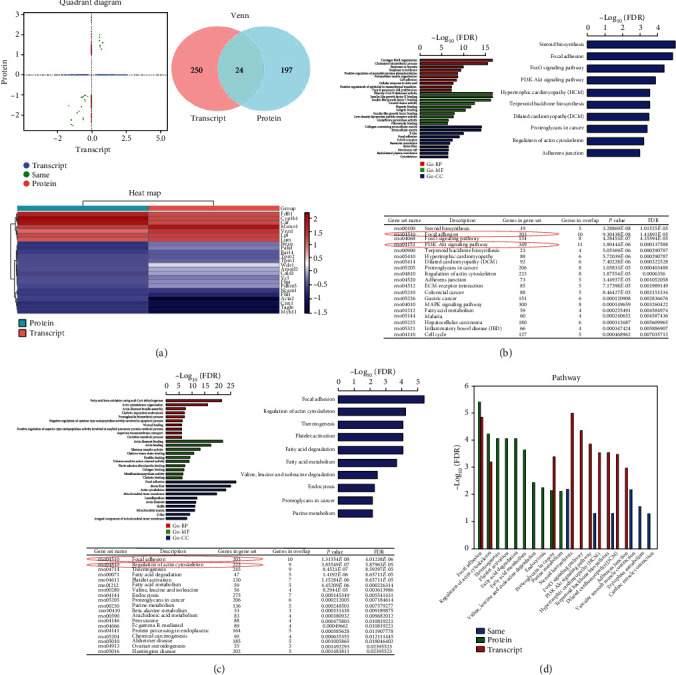
Results of bioinformatics analysis. (a) The quadrant diagram, Venn diagram (Venn), and heatmap of combined analysis of transcriptomics and proteomics. (b) The transcriptome sequencing results, including GO and KEGG enrichment analyses. (c) The proteomic results, including GO and KEGG enrichment analyses. (d) Signaling pathway enrichment analysis of combined transcriptomic and proteomic sequencing results.

To investigate transcriptome and proteome alterations associated with micropattern SF films, we performed high-throughput RNA sequencing and liquid chromatography mass spectrometry proteomics on normal and micropattern SF film-cultured TSPCs. Compared to control cells, TSPCs cultured in micropattern SF films exhibited 250 DEGs and 197 DEPs, of which an overlap of 24 was detected at both the mRNA and protein levels (Figure 4(a)).

Functional enrichment analyses were performed based on the significantly DEGs from RNA sequencing data. Of note, among the 10 most significantly enriched terms in each category of Gene Ontology (GO) enrichment analysis, terms such as ECM organization in biological process (BP) category, integrin binding in molecular function (MF) category, and basement membrane and cytoskeleton in cellular component (CC) category suggested the integrin-mediated cellular remodeling effect of micropattern SF film on the cultured TSPCs. In accordance with the GO analysis results, Kyoto Encyclopedia of Genes and Genomes (KEGG) pathway analysis also showed enrichment of DEGs in regulation of the actin cytoskeleton pathway (Figure 4(b)). Interestingly, there were enriched signaling pathways including FAK and PI3K/AKT, indicating the potential mechanisms underlying the regulation of TSPCs by micropattern SF films.

To gain insights into the cellular functions and biological processes that are affected in TSPCs by micropattern SF film, we also performed GO enrichment analysis of the identified DEPs. Similar to what we found by RNA sequencing, DEPs in TSPCs cultured in micropattern SF films also showed enriched actin cytoskeleton organization term in the BP category, actin binding in the MF category, and focal adhesion and actin cytoskeleton terms in CC categories. KEGG pathway analysis using the DEPs further displayed the top enrichment of focal adhesion and regulation of actin cytoskeleton signaling pathways (Figure 4(c)). Taken together, the combination of KEGG enrichment analysis of DEGs from RNA sequencing and proteins from proteomics suggested that FAK and PI3K/AKT were the major signaling pathways involved in the regulation of TSPCs by micropattern SF films (Figure 4(d)).

### 3.7. Results of Western Blotting and Immunofluorescence

**Figure 5:**
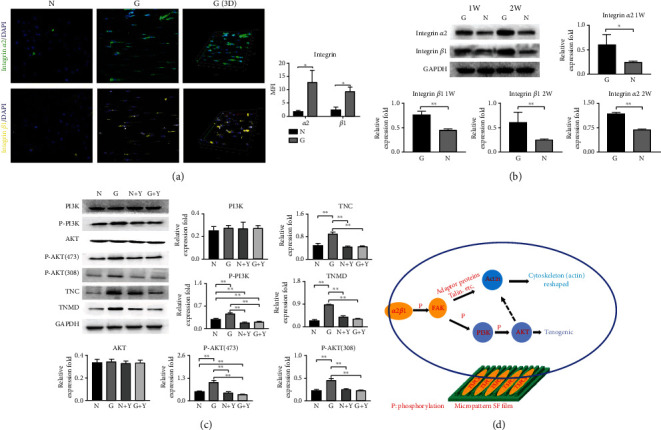
Validation of key proteins in signaling pathways. (a) Immunofluorescence results of *α*2*β*1. The expression level of Group G was significantly higher than that of group N. (b) Western blot analysis of integrin *α*2*β*1. *α*2*β*1 was significantly increased in Group G. (c) Western blot analysis of the PI3K/AKT signaling pathway. The TNC, TNMD, and phosphorylated PI3K/AKT (p-PI3K, p-AKT308, and p-AKT473) were significantly increased in Group G. After adding PI3K/AKT pathway inhibitors, the expression of TNC and TNMD was inhibited simultaneously with phosphorylated PI3K and AKT. (d) Schematic diagram of the signaling pathway of micropattern SF films regulating TSPCs.

As shown in Figures 5(a) and 5(b), Western blotting and immunofluorescence showed that integrins *α*2/*β*1 were significantly increased in Group G, suggesting that *α*2*β*1 were the main cell surface sensors for the regulation of TSPCs by micropattern SF films. As shown in Figure 5(c), TNC, TNMD, and phosphorylated PI3K/AKT (pPI3K, pAKT308, and pAKT473) were significantly increased in Group G. After adding PI3K/AKT pathway inhibitors, the expression of TNC and TNMD was inhibited simultaneously with phosphorylation of PI3K and AKT.

## 4. Discussion

During the healing process of tendons, the infiltration of inflammatory cells leads to edema. In the fat suppression image of the T2 sequence of MRI scans, the inflammatory edema appears bright white, whereas collagen fibers of the normal tendon appear dark black [26, 31–33]. In this study, the MRI results in the micropattern SF film group showed a continuous black stripe signal after 8 months of repair, and the intensity was highest among all experimental groups, indicating that the micropattern SF films performed better in tendon reconstruction. In addition, H&E staining and IHC showed more orderly aligned collagen fibers and higher expression of tendon makers in the regenerated tendons at both 4 and 8 weeks after injury in the micropattern SF film group, suggesting that it is a superior biomaterial in terms of collagen fiber remodeling effect.

Our previous studies demonstrated that the micropattern SF films could change the cell morphology of TSPCs and induce phosphorylation of FAK as well as promote tenogenic differentiation of TSPCs *in vitro* [21, 22]. Integrins are key signal transduction molecules that mediate the regulation of stem cells by bioactive materials. For example, titanium with a rough surface can induce the osteogenic differentiation of MG63 cells through *α*2*β*1 integrin molecules [34]. Integrins can also mediate the adhesion and osteogenic differentiation of osteoblasts. A scaffold made of collagen/glycosaminoglycan can promote the secretion of extrachondral matrix of stem cells, accompanied by the upregulation of integrins *α*2, *α*5, and *β*1 [35]. In addition, Iwamoto and Calderwood [36] showed that integrins can regulate cell adhesion by adjusting their own conformation. We provide evidence showing that integrins *α*2*β*1 specifically mediate the regulation of micropattern SF films in TSPCs, consistent with observations in many previous studies showing that integrins aggregate and change conformation after sensing regulatory signals in biomaterial, leading to the activation of downstream signaling cascades in the cell [37–41].

Although morphological changes have been observed in cultured cells in various types of biological materials, the underlying intrinsic signaling transduction pathway is not fully understood [42–46]. We previously found that TSPCs seeded on the surface of micropattern SF films developed an elongated morphology and directed spatial distribution [21, 22]. Through high-throughput transcriptomic and proteomic assays, this study showed that micropattern SF films promote the expression of talin, paxillin, and actin of FAK/actin pathways in TSPCs. Talin and paxillin are the downstream effectors of integrin in cells and transmit physical stimulating signaling from the ECM to cells, leading to changes in the biological behavior of the seeded cells [47–50]. Therefore, micropatterned SF film can regulate the synthesis of actin through the FAK/actin signaling pathway and thus regulate the morphological changes of TSPCs.

The PI3K/AKT signaling pathway plays a key role in regulating cells under physical stimulation [51]. The signaling pathway was also significantly enriched in the DEGs in RNA sequencing data. Tian et al., Quan et al., Liu et al., and Sun et al. [52–55] found that modulating the cytoskeleton using perturbing drugs changes signal transduction and the differentiation tendency of mesenchymal stem cells. Gu et al. [56] found that the surface-modified titanium matrix can promote the secretion of bone calcium matrix by mesenchymal stem cells through the PI3K/AKT signaling pathway. In addition, Wang et al. [57] found that the three-dimensional uniaxial mechanical stimulation can induce the tenogenic differentiation of TSPCs through the PI3K/AKT signaling pathway. Through blocking experiments, we found that the PI3K/AKT signaling pathway plays a key role in the micropattern SF films-induced tenogenic differentiation of TSPCs. We also found the increased phosphorylation at S308 and S473 of AKT, indicating activation of the downstream mechanistic target of rapamycin complex 1 (mTORC1) and mTORC2 signaling cascades [58, 59]. The biological effects of mTORC1 and mTORC2 are not the same. mTORC1 is mainly involved in gene transcription and protein expression, whereas mTORC2 is mainly involved in the synthesis and remodeling of the cytoskeleton actin [60, 61].

## 5. Conclusions

As summarized in Figure 5(d), in the process of micropattern SF film-regulated TSPCs morphological and functional changes, integrins *α*2*β*1 sense and transmit the stimulating signal into the cells and initiate a series of phosphorylation at the downstream kinases. The *α*2*β*1/FAK/PI3K/AKT/mTORC2 signaling cascade is mainly responsible for the remodeling of actin, which coregulates the remodeling of the cytoskeleton actin with the FAK/actin pathway and induces the morphological changes of TSPCs. Instead, the *α*2*β*1/FAK/PI3K/AKT/mTORC1 signaling cascade regulates gene transcription and translation and promotes the tenogenic differentiation of TSPCs. In conclusion, micropattern SF films have excellent physical performance and stable biological function, it can promote the tendon differentiation of TSPCs *in vitro* and tendon repair *in vivo* through physical signal transmission. It has a promising application prospect in tendon repair, but its achievements transformation and large-scale preparation still need to be further improved.

## Data Availability

The datasets generated and analyzed during the current study are not publicly available but are available from the corresponding authors upon reasonable request.

## Supplementary Materials

Supplementary Materials Table S1: histological scoring system.
