# Supplementary material for: Micropattern Silk Fibroin Film Facilitates Tendon Repair In Vivo and Promotes Tenogenic Differentiation of Tendon Stem/Progenitor Cells through the α2β1/FAK/PI3K/AKT Signaling Pathway In Vitro
Source: Stem Cells Int. 2023 Jan 13;2023:2915826. doi: 10.1155/2023/2915826 (PMC9859702; doi:10.1155/2023/2915826)
Supplement: Supplementary Materials — Table S1: histological scoring system. [file 2915826.f1.docx]

**Table S1** Histological Scoring System

**Fiber Arrangement**

dense, neatly arranged 2

**Evaluation indicators**

**Score**

partially dense, loose or disordered 1

Cluttered and disordered 0

**Number of Cells**

normal 2

increased local cell density 1

abnormal increase of cells and decreased of ECM 0

**Cell Arrangement**

uniaxial arrangement 2

10-50% of cells are irregularly arranged 1 more than 50% cells are irregularly arranged 0

**Cell Distribution**

Uniform and physiological 1

cell clustering 0

**Nuclear Morphology**

Elongated or heterochromatic nucleus 2

large, oval, polymorphic heterochromatin nuclei in 10-30% of nuclei 1 large, oval, euchromatin and heterochromatin nuclei in more than 30% of nuclei 0

**Components of The Scar in Repaired Area**

Homogeneous (one component) 2

Local abnormal tissue composition 1

The entire tissue is completely replaced by other ingredients 0

**Appearance**

Normal form, normal gloss 2

Partial thickening and reduced gloss 1

Significantly thicker, less glossy, less elastic and less rigid 0

| **Evaluation indicators** | **Score** |
| --- | --- |
| **Tissue Metaplasia** |  |
| none | 3 |
| partial swelling | 2 |
| fatty infiltration or scar fibrosis | 1 |
| cartilage or heterotopic ossification  **Neovascularization** | 0 |
| few new capillaries | 1 |
| Significant increase in new blood vessels  **Inflammation** | 0 |
| no inflammatory cells | 1 |
| inflammatory cells infiltration | 0 |
